# Metabolic shifts in plasma amino acids and related metabolites in response to SGLT2 inhibition and hyperglycemia in type 1 diabetes

**DOI:** 10.14814/phy2.70465

**Published:** 2025-08-22

**Authors:** Luxcia Kugathasan, Nagarjunachary Ragi, Subrata Debnath, Vikas S. Sridhar, Soumya Maity, Tianqing Feng, Esmeralda Treviño, Bruce A. Perkins, David Z. I. Cherney, Kumar Sharma

**Affiliations:** ^1^ Division of Nephrology, Department of Medicine University Health Network Toronto Ontario Canada; ^2^ Department of Physiology University of Toronto Toronto Ontario Canada; ^3^ Cardiovascular Sciences Collaborative Specialization University of Toronto Toronto Ontario Canada; ^4^ Center for Precision Medicine University of Texas Health San Antonio San Antonio Texas USA; ^5^ Division of Nephrology, Department of Medicine University of Texas Health San Antonio San Antonio Texas USA; ^6^ Division of Nephrology University of British Columbia Vancouver British Columbia Canada; ^7^ Lunenfeld‐Tanenbaum Research Institute Sinai Health System Toronto Ontario Canada; ^8^ Division of Endocrinology and Metabolism, Department of Medicine University of Toronto Toronto Ontario Canada

**Keywords:** amino acids, empagliflozin, hyperglycemia, metabolites, SGLT2 inhibition, type 1 diabetes

## Abstract

Regulated kidney function is dependent on maintaining efficient energy utilization. Our aim in this study was to determine the effects of acute, ambient hyperglycemia and sodium‐glucose cotransporter‐2 (SGLT2) inhibition on plasma amino acid metabolism in patients with type 1 diabetes (T1D). The ATIRMA trial, a single‐arm study, evaluated the effects of 8 weeks of oral empagliflozin (25 mg/day) in 40 young adults with T1D. The study involved consecutive two‐day assessments of clamped euglycemia and hyperglycemia at both baseline and post‐treatment. MetaboAnalyst 6.0 categorized 35 metabolites into significant pathways, which were statistically compared using principal component analysis. Acute hyperglycemia induced changes to 10 metabolic pathways, including but not limited to increases in cysteine and methionine metabolism (0.52 ± 0.12, *p <* 0.0001), valine, leucine, and isoleucine biosynthesis (0.31 ± 0.10, *p* = 0.002); and nitrogen metabolism (0.11 ± 0.03, *p* = 0.003). Introduction of empagliflozin was associated with a decrease in adenine, and an increase in cysteine and methionine metabolism (0.31 ± 0.13, *p* = 0.02) when maintained under euglycemia and a decrease in nitrogen metabolism under hyperglycemia (−0.07 ± 0.04, *p* = 0.04). Our findings show that SGLT2 inhibition counteracts the hyperglycemia‐induced changes in plasma amino acid metabolism, potentially improving energy efficiency and metabolic health, though more research is needed to confirm these metabolic effects.

## 1 INTRODUCTION

The glycemic benefits of sodium‐glucose cotransporter‐2 (SGLT2) inhibitors in individuals with type 1 diabetes (T1D) have been consistently reported in the few randomized controlled trials conducted to date, namely the DEPICT (Dandona et al., 2017, 2018; Mathieu et al., 2018, 2020), EASE (Rosenstock et al., 2018), and inTandem (Buse et al., 2018; Danne et al., 2018; Garg et al., 2017; Sridhar et al., 2023) trials. However, growing safety concerns, particularly regarding the heightened risk of diabetic ketoacidosis (DKA) (Groop et al., 2020; Phillip et al., 2021; Taylor et al., 2019), have raised questions about their long‐term use, leading to a reconsideration of their role in T1D glycemic management. SGLT2 inhibitors have proven cardiovascular and kidney benefits in patients with type 2 diabetes (T2D) (Heerspink et al., 2016, 2018; Zinman et al., 2015). Considering the substantial residual risk for cardiovascular disease and kidney failure in individuals with T1D despite guideline‐directed therapy (Khunti et al., 2015; Lee et al., 2019), the use of SGLT2 inhibitors may improve the overall benefit–risk balance in patients with T1D.

The development of cardiovascular and kidney events follows similar pathophysiological pathways in both T1D and T2D (Cherney, Perkins, Soleymanlou, Maione, et al., 2014; Mulder et al., 2019). Emphasizing the common pathways targeted by SGLT2 inhibitors provides a strong rationale for their clinical investigation and potential use in T1D management. Impaired amino acid metabolism is a distinct metabolic hallmark of diabetes, likely developed as a consequence of insulin resistance, changes in protein turnover, and kidney dysfunction (Knol et al., 2024). This bioenergetic mismatch accelerates a pathological shift in mitochondrial substrate utilization, affecting multiple organ systems (Zhu et al., 2022). It is hypothesized that a key metabolic effect of SGLT2 inhibition is the induction of a “pseudo‐fasting” state, where increased glucosuria drives a shift in metabolism from glucose utilization to the enhanced use of circulating amino acids, ketones, and lipids for energy (Ferrannini, Baldi, et al., 2016; Santos‐Gallego et al., 2019). This metabolic shift suggests that SGLT2 inhibition modulates mitochondrial energetics by enhancing the utilization of amino acid substrates at a systemic level (Ferrannini, Mark, & Mayoux, 2016). Moreover, many nonamino acid metabolites (e.g., adenine), which are intermediates or byproducts of pathways linked to amino acid catabolism (Badawy, 2017; Oxenkrug, 2013), accompany the changes in amino acid levels due to SGLT2 inhibition (Sharma et al., 2023), particularly in processes related to energy production and cellular function (Kugathasan et al., 2024). Whether this metabolic benefit of SGLT2 inhibition extends to patients with T1D is unknown.

Our current analysis evaluated the changes in individual and grouped plasma amino acid levels and related metabolites in a cohort of patients with T1D in response to (1) hyperglycemia, (2) SGLT2 inhibitor empagliflozin under clamped euglycemia, and (3) empagliflozin under clamped hyperglycemia. We hypothesized that SGLT2 inhibition will attenuate any increase in plasma metabolite levels in response to hyperglycemia.

## 2 MATERIALS AND METHODS

### 2.1 Research design and study participants

We performed a secondary exploratory analysis of the 8‐week Adjunctive‐To‐Insulin and Renal Mechanistic (ATIRMA) trial (NCT01392560) (Cherney, Perkins, Soleymanlou, Maione, et al., 2014) to examine changes in plasma metabolites associated with empagliflozin and acute hyperglycemia. The ATIRMA study was an open‐label, single‐arm, mechanistic trial with the primary objective to evaluate the physiological effects of 8 weeks of 25 mg empagliflozin on glomerular hyperfiltration in 40 young adults with T1D who were normotensive and normoalbuminuric (Cherney & Perkins, 2014; Cherney, Perkins, Soleymanlou, Har, et al., 2014; Kopecky et al., 2019; Perkins et al., 2014, 2015; van Bommel et al., 2020). Of the 40 patients, 27 were classified as hyperfiltering (with an estimated glomerular filtration rate [eGFR] ≥ 135 mL/min/1.73 m^2^) (Cherney et al., 2009), while the remaining 13 were considered normofiltering (with an eGFR of 90–134 mL/min/1.73 m^2^). Of the 40 participants, 20 were female and 20 were male. Patient characteristics for this cohort are published elsewhere (Cherney, Perkins, Soleymanlou, Maione, et al., 2014).

**FIGURE 1:**
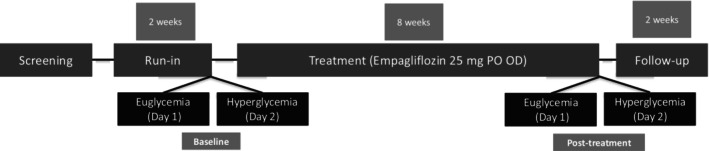
Study design for the ATIRMA trial. Empagliflozin 25 mg oral once daily was administered for 8 weeks to 40 participants with T1D.

The ATIRMA trial was structured into three phases: a 2‐week placebo run‐in period, an 8‐week treatment phase with a daily dose of 25 mg of empagliflozin, and a 2‐week follow‐up period (as depicted in Figure 1). Boehringer Ingelheim Ltd. Canada was the supplier of empagliflozin drug. After the run‐in and treatment phases, glycemic exposure was assessed over two consecutive days: Day 1 involved clamped euglycemia (plasma glucose of 4–6 mmol/L), and Day 2 involved clamped hyperglycemia (plasma glucose of 9–11 mmol/L). To mitigate the risk of hypoglycemia associated with empagliflozin, both prandial and basal insulin doses were initially reduced by 30%. This initial reduction was subsequently adjusted under the guidance of the investigator, resulting in an average decrease of 16.4% (Cherney, Perkins, Soleymanlou, Maione, et al., 2014).

### 2.2 Measurement of plasma amino acids and related metabolites

Amino acids and related metabolites were measured from plasma samples by bulk liquid chromatography (LC) and mass spectroscopy (MS) analysis (Table S1). The plasma (10 μL) samples were transferred into a fresh 96‐well plate and extracted with 80% methanol. The plate was vortexed for 1 min, followed by centrifuging the samples for 10 minutes at 9000 RPM at 4°C. Next, 5 μL of supernatant was transferred into a 96‐well plate for LC/MS analysis. Analysis was performed on a Thermo Q Exactive HF‐X Orbitrap mass spectrometer (Thermo, San Jose, CA) interfaced with a heated electrospray ionization source (HESI) and coupled with a Thermo Vanquish HPLC system. An aliquot of 2.5 μL of the sample was injected into the instrument using an autosampler. The chromatographic separation took place on an Agilent ZORBAX HILIC PLUS column with a 3.5 μm particle size and with dimensions of 2.1 × 100 mm with a phase composition of 10 mM ammonium formate, 0.05% formic acid in Millipore water (component A), and 0.05% formic acid in acetonitrile (component B) at a 0.3 mL/ min flow rate (Pallerla et al., 2023). Data acquisition and data processing was carried out using Thermo Xcalibur Quant Browser software version 4.2.47.

Retention time of metabolites was tabulated in the Table S2, and for the assignment of metabolite identities, commercially available pure standards (≥90%–99% purity; primarily from Sigma‐Aldrich and MedChemExpress) were analyzed under identical LC–MS conditions as the experimental samples. Standards included, but were not limited to, nicotinic acid, 5‐methylthioadenosine, SAM, SAH, ADMA, SDMA, MMA, GABA, and a comprehensive amino acid mix (MSK‐A2‐US‐1.2, CIL Inc.); full details including CAS numbers, vendors, and purities are provided in Table S3. Metabolite identities were confirmed by matching the retention time (±0.05 min) and accurate mass (±5 ppm) to those of the standards. MS/MS fragmentation spectra were acquired for selected metabolites and compared to spectra from reference standards. Retention times and signal intensities of internal standards and quality control samples were monitored across runs to ensure consistency. Any drift in retention time or changes in peak shape triggered manual review of the assignment. Peak integration was checked and adjusted as needed to maintain accuracy in quantification. By combining accurate mass, retention time, and MS/MS fragmentation, the approach ensures robust metabolite identification and facilitates reproducibility of results.

### 2.3 Mapping amino acid metabolic networks

MetaboAnalyst 6.0 software (Pang et al., 2024) was employed to classify 19 plasma amino acids and 14 related metabolites into physiologically relevant metabolic pathways. Pathway analysis was conducted according to standardized human metabolic pathways from the Kyoto Encyclopedia of Genes and Genomes (KEGG) database. To evaluate the likelihood of random occurrences within specific metabolic pathways, we performed an overrepresentation analysis using hypergeometric distributions. A false discovery rate (FDR) threshold of ≤0.1 was established to select pathways that were significantly represented by the metabolites.

Thirty‐five measured plasma metabolites were grouped into 13 metabolic pathways by MetaboAnalyst 6.0: cysteine and methionine metabolites; arginine metabolites; valine, leucine, and isoleucine metabolites; alanine, aspartate, and glutamate metabolites; arginine and proline metabolites; glyoxylate and dicarboxylate metabolites; glycine, serine, and threonine metabolites; one carbon pool by folate metabolites; glutathione metabolism; taurine and hypotaurine metabolism; phenylalanine, tyrosine, and tryptophan biosynthesis; phenylalanine metabolism; and nitrogen metabolites (Table S4).

To reduce dimensionality, principal component analysis (PCA) was used to project the metabolites from each pathway onto the first principal component, resulting in Eigenvalues that indicated the contribution of each metabolite group to their respective pathways. It is noteworthy that the phenylalanine metabolic pathway and the phenylalanine, tyrosine, and tryptophan biosynthesis metabolic pathway contained identical amino acid compositions, leading to a combined PCA for both pathways.

### 2.4 Statistical analyses

We conducted linear regression analyses for repeated measures to evaluate changes in individual plasma metabolite levels and grouped metabolites following PCA. The results were expressed as Z‐scores derived from linear mixed‐effects models. We specifically examined differences across four conditions: baseline clamped euglycemia, baseline clamped hyperglycemia, post‐treatment clamped euglycemia, and post‐treatment clamped hyperglycemia. This was done using two‐tailed hypothesis testing based on a least squares means model with an unstructured random‐effects covariance matrix for the intercept and independent errors. Each visit was treated as a repeated measure for the respective subject.

To enhance the normal distribution of residuals and mitigate skewness, the data underwent log transformation before PCA. To analyze the interaction effects of empagliflozin treatment and acute hyperglycemia, we conducted an interaction analysis for both individual and grouped metabolites. Changes in individual plasma amino acids and grouped pathways with empagliflozin and acute hyperglycemia were reported as differences in least squares means estimates of log‐transformed values. Two‐tailed hypothesis testing was utilized at a significance level of 5% to compare the treatment effects on grouped metabolites. For the individual metabolites, we applied the Benjamini‐Hochberg procedure to correct for multiple comparisons, assuming independence and defining significant results as an FDR of ≤0.05. The adjusted *p* values from the Benjamini‐Hochberg procedure are reported. This approach was chosen to reduce the likelihood of false positives in our exploratory analysis of individual metabolites. All analyses were carried out using SAS System Version 9.4 (SAS Institute, Cary, NC).

## 3 RESULTS

### 3.1 Baseline characteristics

The baseline characteristics of the ATIRMA trial have been previously reported (Cherney, Perkins, Soleymanlou, Maione, et al., 2014; Perkins et al., 2014). This secondary analysis included 27 individuals with hyperfiltration (estimated glomerular filtration rate [eGFR] ≥ 135 mL/min/1.73m^2^) and 13 with normofiltration (eGFR 90–134 mL/min/1.73m^2^), all with T1D. The mean age of participants was 24.3 ± 5.1 years, with a mean diabetes duration of 17.1 ± 7.1 years (Perkins et al., 2015). The cohort was equally distributed by sex and had a mean body‐mass index of 24.5 ± 3.2 kg/m^2^. None were taking renin‐angiotensin system blockers, statins, or metformin.

### 3.2 Plasma amino acid metabolism in response to acute hyperglycemia

**FIGURE 2:**
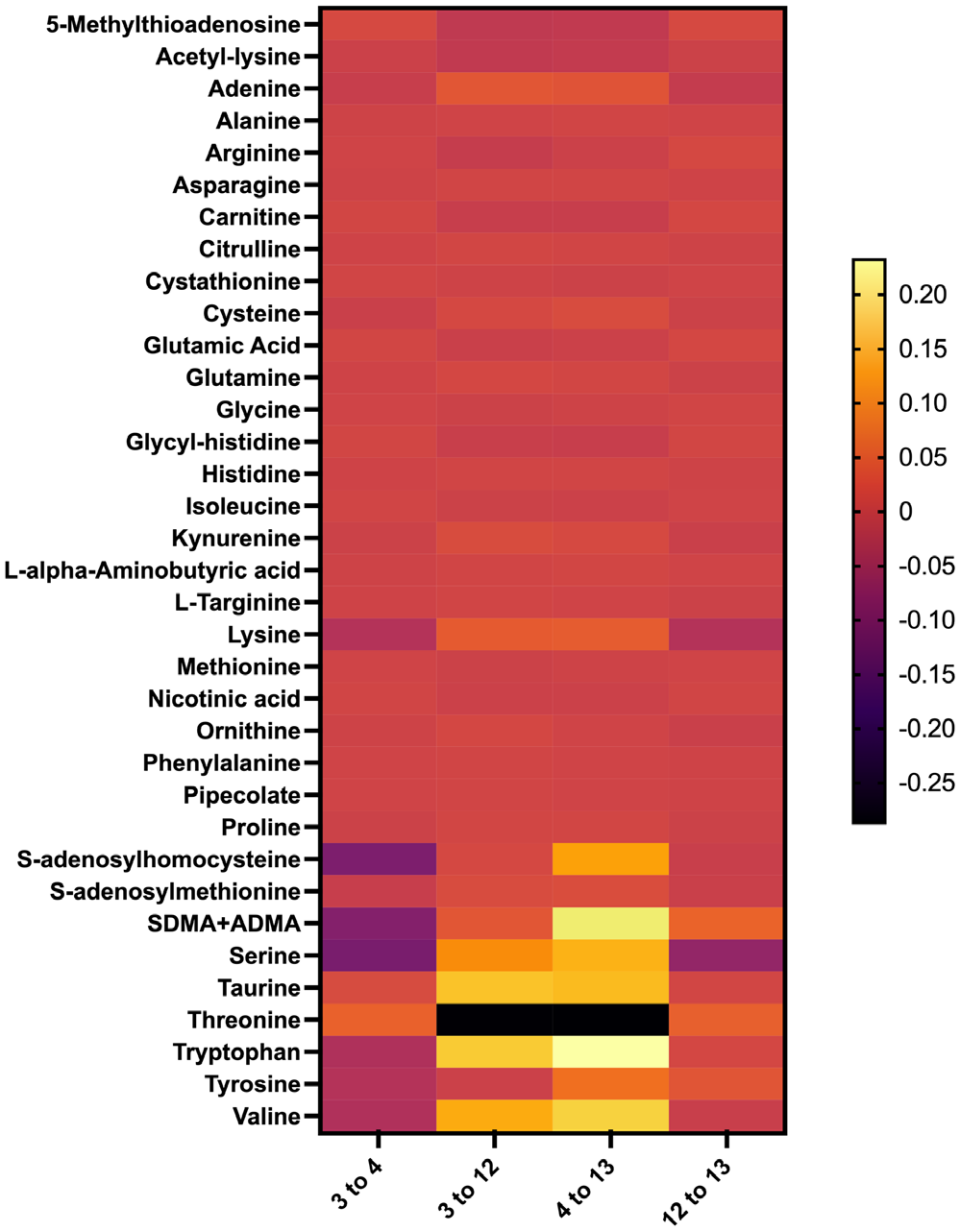
Heat map of plasma metabolites significantly changed by hyperglycemia and empagliflozin. 3–4—baseline, clamped euglycemia versus clamped hyperglycemia; 3–12—clamped euglycemia, baseline versus post‐treatment; 4–13—clamped hyperglycemia, baseline versus post‐treatment; 12–13—post‐treatment, clamped euglycemia versus clamped hyperglycemia. Two‐tailed hypothesis testing was utilized at a significance level of 5% to compare the treatment effects on metabolites. For the individual metabolites, we applied the Benjamini‐Hochberg procedure to correct for multiple comparisons, assuming independence and defining significant results as an FDR of ≤0.05.

Under acute hyperglycemia, the level of six individual amino acids was significantly altered compared to euglycemic levels (Figure 2). Specifically, hyperglycemia was associated with decreases in methionine (−0.20, *p* = 0.004), serine (−0.14, *p* = 0.004), threonine (−0.15, *p* = 0.01), glutamine (−0.07, *p* = 0.02), proline (−0.15, *p* = 0.03), and alanine (−0.19, *p* = 0.004) (Table 1).

**TABLE 1:** Change in least square means estimate of log transformed plasma metabolites between euglycemia and acute hyperglycemia at baseline. Ranked by Benjamini Hochberg adjusted *p* values.

Metabolite	Euglycemia versus hyperglycemia, baseline (3, 4)	
	Estimate	*p* Value
Alanine	−0.1853	0.0035
Methionine	−0.1996	0.0035
Serine	−0.1402	0.0035
Threonine	−0.1495	0.0105
Glutamine	−0.0737	0.0245
Proline	−0.1473	0.0289
Asparagine	−0.0919	0.0588
Taurine	0.1152	0.0858
Kynurenine	−0.0251	0.0858
Arginine	−0.1151	0.0858
Adenine	−0.2651	0.3002
Lysine	−0.0529	0.3157
L‐alpha‐Aminobutyric acid	−0.0852	0.3157
Tryptophan	−0.0387	0.4066
Tyrosine	−0.2472	0.4440
Isoleucine	−0.1048	0.5650
Glycyl‐histidine	0.1515	0.5650
Acetyl‐Lysine	0.0520	0.5650
Glycine	−0.0467	0.5650
Glutamic acid	0.0718	0.5650
Ornithine	−0.0626	0.5650
S‐adenosylmethionine	0.0123	0.5650
Carnitine	0.0292	0.5650
SDMA+ADMA	0.0960	0.5732
Cysteine	0.0709	0.5732
Cystathionine	0.0941	0.5732
Histidine	−0.0164	0.6077
Nicotinic acid	0.0699	0.7035
Phenylalanine	−0.0407	0.7684
S‐adenosylhomocysteine	0.1651	0.7748
5‐Methylthioadenosine	−0.1225	0.7748
Mono‐Methyl Arginine	−0.0248	0.8597
Citrulline	−0.0124	0.8936
Pipecolate	−0.0146	0.8936
Valine	−0.0076	0.9030

*Note*: 3–4—baseline, clamped euglycemia versus clamped hyperglycemia. All *p* values were adjusted with Benjamini Hochberg multiple comparisons.

**FIGURE 3:**
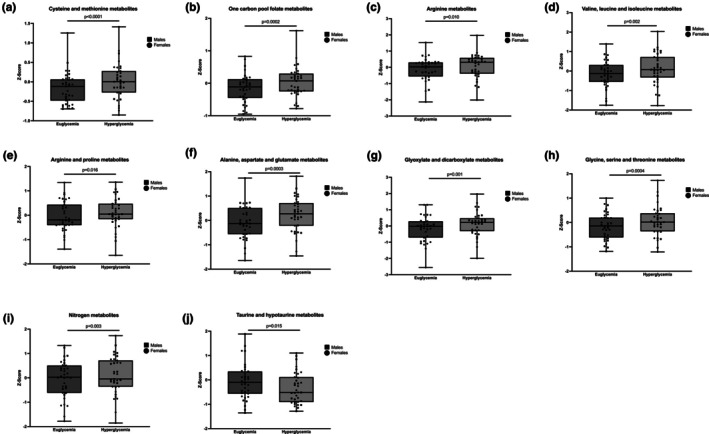
Plasma metabolite pathways altered by acute hyperglycemia at baseline is reflected as a Z‐score of mean ± standard deviations. (a) Cysteine and methionine metabolites, (b) one carbon pool by folate metabolites, (c) arginine metabolites, (d) valine, leucine, and isoleucine metabolites, (e) arginine and proline metabolites, (f) alanine, aspartate, and glutamate metabolites, (g) glyoxylate and dicarboxylate metabolites, (h) glycine, serine, and threonine metabolites, (i) nitrogen metabolites, and (j) taurine and hypotaurine metabolites. Metabolomic pathway compositions were identified by MetaboAnalyst 6.0 software to accurately group 35 metabolites measured in plasma in this post hoc analysis with an FDR ≤ 0.1 (Table S1). Metabolites from each pathway were projected onto first principal component using PCA to represent the pathway composition (Table S4). Repeated measures linear regression and post hoc least squares mean estimates were performed. Significant *p* values are stated.

Of the grouped pathways identified using the 19 available plasma amino acids and 14 related metabolites, hyperglycemia significantly changed 10 out of the 12 pathways. This includes increases in cysteine and methionine metabolism; one carbon pool by folate; arginine biosynthesis; valine, leucine, and isoleucine biosynthesis; arginine and proline metabolism; alanine, aspartate, and glutamate metabolism; glyoxylate and dicarboxylate metabolism; glycine, serine, and threonine metabolism; and nitrogen metabolism, as well as decreases in taurine and hypotaurine metabolism (Figure 3; Table 2).

**TABLE 2:** Least square means change in grouped plasma metabolites levels for comparison between euglycemia and hyperglycemia timepoints. Data reported as mean ± standard deviations.

Group	3–4
Cysteine and methionine metabolism	0.52 ± 0.12 *p <* 0.0001
One carbon pool by folate	0.53 ± 0.14 *p* = 0.0002
Arginine biosynthesis	0.34 ± 0.13 *p* = 0.0104
Valine, leucine, and isoleucine biosynthesis	0.31 ± 0.10 *p* = 0.0021
Arginine and proline metabolism	0.27 ± 0.11 *p* = 0.0160
Alanine, aspartate, and glutamate metabolism	0.49 ± 0.13 *p* = 0.0003
Glutathione metabolism	0.11 ± 0.11 *p* = 0.2848
Glyoxylate and dicarboxylate metabolism	0.43 ± 0.13 *p* = 0.0012
Phenylalanine, tyrosine, and tryptophan biosynthesis	0.02 ± 0.01 *p* = 0.0705
Glycine, serine, and threonine metabolism	0.48 ± 0.13 *p* = 0.0004
Nitrogen metabolism	0.11 ± 0.03 *p* = 0.0026
Taurine and hypotaurine metabolism	−0.14 ± 0.06 *p* = 0.0149

*Note*: 3–4—baseline, clamped euglycemia versus clamped hyperglycemia.

### 3.3 Plasma amino acid metabolism in response to SGLT2 inhibition under euglycemia

**FIGURE 4:**
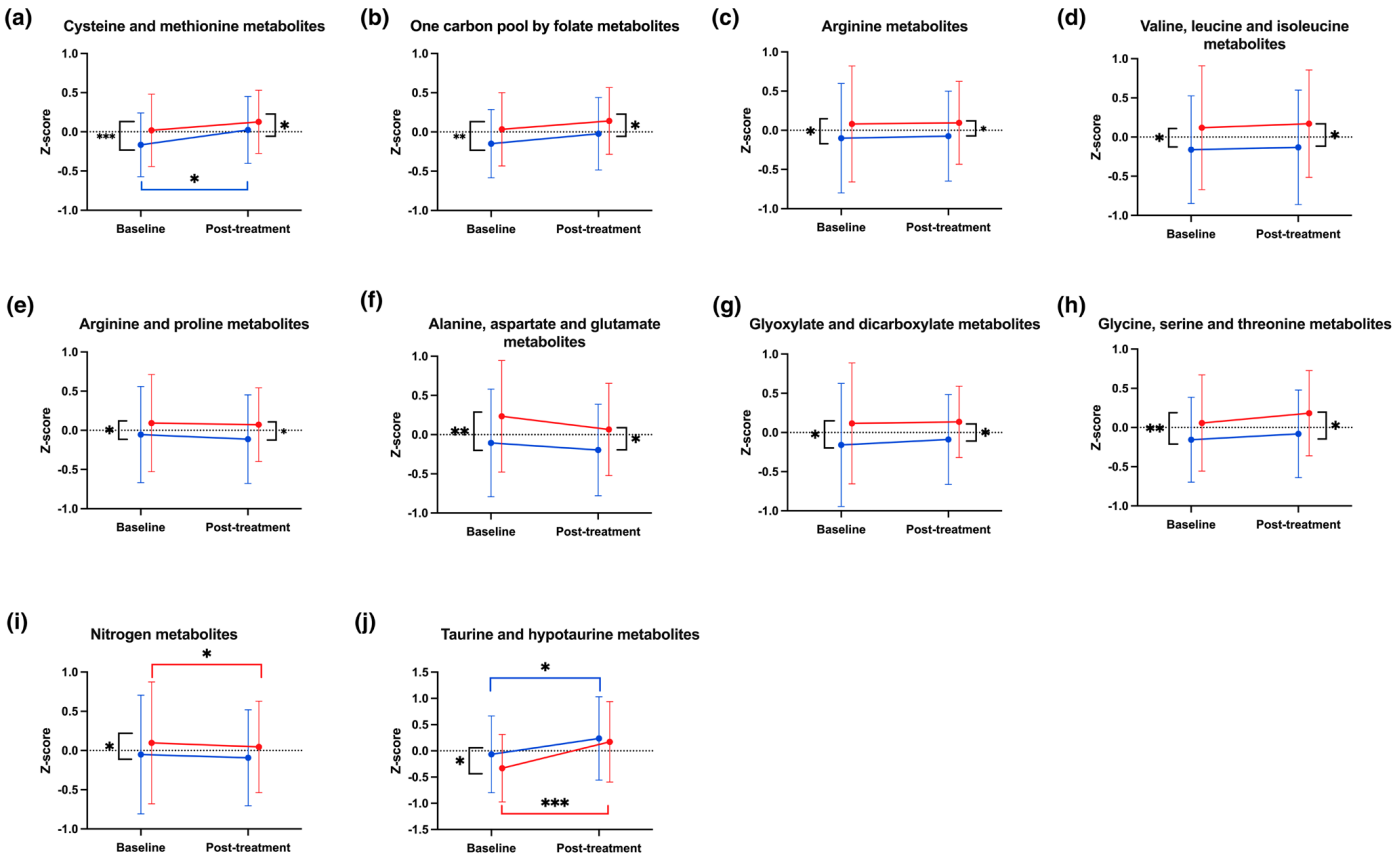
Plasma metabolite pathways at baseline and post‐treatment (8 weeks of 25 mg empagliflozin once daily) are reflected as a Z‐score of mean ± standard deviations. This is under both clamped euglycemic (blue) and hyperglycemia (red). (a) Cysteine and methionine metabolites, (b) one carbon pool by folate metabolites, (c) arginine metabolites, (d) valine, leucine, and isoleucine metabolites, (e) alanine, aspartate, and glutamate metabolites, (f) arginine and proline metabolites, (g) glyoxylate and dicarboxylate metabolites, (h) glycine, serine, and threonine metabolites, (i) nitrogen metabolites, and (j) taurine and hypotaurine metabolites. Metabolomic pathway compositions were identified by MetaboAnalyst 6.0 software to accurately group 35 metabolites measured in plasma in this post hoc analysis with an FDR ≤ 0.1 (Table S1). Metabolites from each pathway were projected onto first principal component using PCA to represent the pathway composition (Table S4). Repeated measures linear regression and post hoc least squares mean estimates were performed. A treatment (T_x_) by glycemia interaction was analyzed for each metabolic pathway. Only significant *p* values are stated. *p ≤ 0.05, **p ≤ 0.001, and ***p ≤ 0.0001.

Of the 35 measured plasma metabolites, empagliflozin under clamped euglycemia significantly changed the levels of six individual amino acids and adenine metabolite (Figure 2). Specifically, empagliflozin was associated with increases in alanine (0.14, *p* = 0.03), kynurenine (0.07, *p* = 0.03), and mono‐methyl arginine (0.40, *p* = 0.004) as well as decreases in L‐alpha‐aminobutyric acid (−0.26, *p* = 0.004), citrulline (−0.15, *p* = 0.03), and acetyl‐lysine (−0.12, *p* = 0.04) (Table 3). Empagliflozin was also associated with a decrease in adenine (−0.43, *p* = 0.04). Under clamped euglycemia, empagliflozin was associated with significant decreases in taurine and hypotaurine metabolism and significant increases in cysteine and methionine metabolism (Table 4; Figure 4).

**TABLE 3:** Change in least square means estimate of log transformed plasma metabolites with empagliflozin treatment. Ranked by Benjamini Hochberg adjusted *p* values.

Metabolite	Baseline versus post‐treatment, at euglycemia (3–12)		Baseline versus post‐treatment, at hyperglycemia (4–13)		Treatment by glycemia
	Estimate	*p* Value	Estimate	*p* Value	Interaction
Adenine	−0.4263	0.0378	0.1312	0.6580	0.0127
Carnitine	0.0257	0.5588	−0.0467	0.2755	0.0895
Tyrosine	0.0329	0.1828	0.0124	1.0000	0.1247
Phenylalanine	−0.0591	0.3589	0.0062	0.0123	0.2199
Cysteine	−0.1512	0.2044	−0.2851	0.0088	0.2377
Acetyl‐Lysine	−0.1213	0.0379	−0.1935	0.0035	0.249
L‐alpha‐Aminobutyric acid	−0.2566	0.0035	−0.1820	0.0088	0.3068
Glutamine	0.0131	0.7798	0.0468	0.1461	0.3189
Asparagine	−0.0469	0.3747	−0.0001	1.0000	0.341
Cystathionine	−0.1353	0.4373	−0.2844	0.0455	0.3467
SDMA+ADMA	−0.1539	0.3698	−0.2948	0.0368	0.3509
5‐Methylthioadenosine	−0.07712	0.8701	0.2647	0.5865	0.3663
Glutamic acid	0.0236	0.8408	−0.0584	0.6580	0.3827
Glycyl‐histidine	0.1773	0.3747	0.0225	1.0000	0.3915
Serine	−0.0383	0.5049	0.0021	1.0000	0.4258
Nicotinic acid	−0.1211	0.5049	0.0023	1.0000	0.4323
Arginine	0.0261	0.7798	0.0783	0.2755	0.4516
Mono‐Methyl Arginine	0.4031	0.0035	0.3242	0.0035	0.4577
Valine	0.0076	0.9321	−0.0346	0.6580	0.4695
Methionine	−0.0163	0.8370	0.0250	0.7643	0.4832
Tryptophan	0.0485	0.2044	0.0732	0.0368	0.5098
Ornithine	0.0437	0.6468	−0.0106	1.0000	0.5199
Pipecolate	−0.01628	0.0580	−0.1053	0.2665	0.524
Taurine	−0.0892	0.2044	−0.1294	0.0368	0.5462
Proline	−0.0025	0.9876	−0.0388	0.6617	0.6065
Kynurenine	0.0715	0.0280	0.1548	0.0455	0.6316
Lysine	−0.0441	0.3747	−0.0244	0.6617	0.6574
S‐adenosylhomocysteine	−0.1803	0.7798	−0.0266	1.0000	0.6643
S‐adenosylmethionine	0.0122	0.5049	0.0186	0.2755	0.707
Alanine	0.1379	0.0280	0.1606	0.0088	0.7254
Histidine	0.0215	0.5049	0.0296	0.3381	0.787
Threonine	0.1051	0.0669	0.0979	0.0751	0.9055
Isoleucine	−0.1403	0.9321	−0.1106	1.0000	0.9439
Glycine	−0.0452	0.5049	−0.0470	0.5277	0.9771
Citrulline	−0.1482	0.0280	−0.1497	0.0140	0.9825

*Note*: 3–12—clamped euglycemia, baseline versus post‐treatment. 4–13—clamped hyperglycemia, baseline versus post‐treatment. All visit change *p* values were adjusted with Benjamini Hochberg multiple comparisons. Interaction—*p* value for treatment by glycemia interaction.

**TABLE 4:** Least square means change in grouped plasma metabolite levels for comparison between timepoints. Data reported as mean ± standard deviation.

Group	3–12	4–13	Interaction
Cysteine and methionine metabolism	0.31 ± 0.13 *p =* 0.0152	0.20 ± 0.13 *p* = 0.1155	0.5364
One carbon pool by folate	0.16 ± 0.14 *p =* 0.2561	0.05 ± 0.14 *p =* 0.7237	0.5804
Arginine biosynthesis	0.01 ± 0.13 *p* = 0.9599	0.10 ± 0.13 *p* = 0.4473	0.6168
Valine, leucine and isoleucine biosynthesis	0.11 ± 0.10 *p* = 0.2715	0.09 ± 0.10 *p* = 0.3657	0.8950
Arginine and proline metabolism	−0.06 ± 0.11 *p =* 0.5686	−0.004 ± 0.11 *p* = 0.9726	0.6700
Alanine, aspartate, and glutamate metabolism	−0.06 ± 0.13 *p* = 0.6680	−0.25 ± 0.13 *p* = 0.0631	0.3118
Glutathione metabolism	−0.02 ± 0.11 *p =* 0.8423	−0.11 ± 0.11 *p* = 0.3126	0.5670
Glyoxylate and dicarboxylate metabolism	0.08 ± 0.13 *p =* 0.5160	0.05 ± 0.13 *p =* 0.7238	0.4806
Phenylalanine, tyrosine, and tryptophan biosynthesis	−0.01 ± 0.01 *p* = 0.3659	0.002 ± 0.01 *p* = 0.8470	0.4406
Glycine, serine, and threonine metabolism	0.06 ± 0.13 *p* = 0.6439	0.01 ± 0.13 *p =* 0.9462	0.7807
Nitrogen metabolism	−0.005 ± 0.04 *p =* 0.8974	−0.07 ± 0.04 *p =* 0.0399	0.1755
Taurine and hypotaurine metabolism	0.16 ± 0.06 *p =* 0.0070	0.26 ± 0.06 *p* < 0.0001	0.2017

*Note*: 3–12—clamped euglycemia, baseline versus post 8‐week treatment. **4–13**—clamped hyperglycemia, baseline versus post 8‐week treatment. Interaction—*p* value for Treatment by glycemia interaction.

### 3.4 Plasma amino acid metabolism in response to SGLT2 inhibition under hyperglycemia

Empagliflozin under clamped hyperglycemia significantly changed the levels of 12 individual amino acids (Table 3). Specifically, empagliflozin was associated with increases in phenylalanine (0.006, *p* = 0.01), tryptophan (0.07, *p* = 0.04), kynurenine (0.15, *p* = 0.046), mono‐methyl arginine (0.32, *p* = 0.004), and alanine (0.16, *p* = 0.009), whereas decreases in cysteine (−0.29, *p* = 0.009), acetyl‐lysine (−0.19, *p* = 0.004), symmetric dimethylarginine (SDMA), and asymmetric dimethylarginine (ADMA) (−0.29, *p* = 0.04), citrulline (−0.15, *p* = 0.01), taurine (−0.13, *p* = 0.04), cystathionine (−0.28, *p* = 0.046), and L‐alpha‐Aminobutyric acid (−0.18, *p* = 0.009) were observed. Moreover, of the 35 individual metabolites, adenine was identified to have a significant treatment by glycemia interaction where the effect of empagliflozin on adenine was modified by hyperglycemia (Table 3). Under clamped hyperglycemia, empagliflozin was associated with significant increases in nitrogen metabolism and taurine and hypotaurine metabolism (Figure 4).

## 4 DISCUSSION

Disruptions to amino acid metabolism play a key role in the development of cardiorenal complications associated with diabetes (Zhu et al., 2022). In the post hoc analysis of the ATIRMA trial, we found that acute, ambient hyperglycemia increased amino acid metabolism, specifically valine, leucine, and isoleucine biosynthesis; alanine, aspartate, and glutamate metabolism; arginine biosynthesis; and arginine and proline metabolism in patients with T1D. Under euglycemia, patients treated with 8 weeks of empagliflozin treatment reported increases in taurine and hypotaurine metabolism and cysteine and methionine metabolism, whereas under acute hyperglycemia, empagliflozin was associated with decreases in nitrogen metabolism. Despite initial increases to amino acid metabolism pathways with acute hyperglycemia, empagliflozin treatment mediated significant changes in plasma metabolites related to energy metabolism and mitochondrial function, implying shifts in metabolic pathways and substrate utilization in the context of T1D.

Branched chain amino acids (BCAAs) are protein‐building groups of amino acids consisting of valine, leucine, and isoleucine. We demonstrate that acute, ambient hyperglycemia in patients with T1D increased BCAA and decreased alanine levels. BCAAs are involved in various cellular signaling pathways, both anabolic and catabolic, that can lead to changes in cellular function and organismal phenotype (Wanders et al., 2012). Notably, BCAAs are recognized for their role in enhancing protein synthesis by regulating protein translation. However, increased levels of plasma BCAAs have been linked to severe insulin resistance (Wang et al., 2011). It is postulated that this increase in BCAAs may stem from impaired BCAA degradation processes, contributing to the development and progression of insulin resistance (Gannon et al., 2018). Moreover, given that T1D is characterized by increased amino acid oxidation, it is proposed that when reduction–oxidation balance is disrupted, the influx of BCAAs from dietary sources and muscle protein breakdown overwhelms the capacity of the body to metabolize them (Holeček, 2020). This results in a significant elevation of plasma BCAA levels in patients with T1D and inadequately controlled glycemic levels (Berger et al., 1978; Borghi et al., 1985; Carlsten et al., 1966). The primary acceptor of the amino nitrogen from BCAAs is α‐ketoglutarate, which is subsequently converted into glutamate. This conversion is accompanied by the conversion of pyruvate to alanine. When BCAA metabolism and conversion to α‐ketoglutarate are disrupted in patients with T1D, alanine levels decrease. Accordingly, our findings are consistent with the hypothesis that suggests elevated BCAA levels in T1D arise from disruptions in glycolysis and fatty acid oxidation due to hyperglycemia, which lead to insufficient BCAA catabolism in muscles (Harper et al., 1984).

Introduction of 8 weeks of empagliflozin therapy in a state of hyperglycemia attenuated the increase in cysteine and methionine metabolism observed under euglycemia. Despite evidence from at least one other analysis validating this finding (Nakano et al., 2020), the resulting physiological implications in the kidney and in circulation, especially in patients with T1D, are not well known. Specifically, in people with diabetes, methionine is reported to have a significantly inverse relationship with hemoglobin A1c (Zhang et al., 2023), suggesting that any increase in methionine and its metabolism should be beneficial for patients with T1D. However, amino acids, such as methionine (Aledo et al., 2011) and cysteine (Moosmann, 2011; Moosmann et al., 2020), have evolved to enhance their resistance to oxidation by modulating the content of sulfur‐containing residues that reflect the reduction–oxidation status of the body. Therefore, adaptive reductions in methionine and cysteine levels have evolved as mechanisms in long‐lived animal species. Beyond their structural and functional roles in proteins, methionine and cysteine participate in complex methionine metabolism (Parkhitko et al., 2019; Sanderson et al., 2019). As such, emerging evidence suggests that regulation of methionine metabolism may play a critical role in determining longevity (Mota‐Martorell et al., 2022), supporting our findings that the attenuation in the increase in cysteine and methionine metabolism associated with empagliflozin under hyperglycemic conditions may be protective. Additional research is needed to clarify this potential mechanism.

As highlighted in our previous findings across several patient cohorts, urine adenine may potentially serve as a mechanistic biomarker for end‐stage kidney disease (Sharma et al., 2023). Specifically, patients with the highest tertile of urine adenine‐to‐creatinine ratio were associated with an increased risk of kidney failure and mortality. Notably, however, we have previously shown that empagliflozin effectively reduced elevated urine adenine‐to‐creatinine levels in patients with T1D in the ATIRMA trial by 36% (from 70.9 to 44.8 nM/mM) (Sharma et al., 2023). In our current analysis, we found that plasma adenine levels decreased with empagliflozin therapy by 13% (from 6.8 to 5.9 nM); however, this was exclusively under an euglycemic clamp, as corroborated by the significant treatment by glycemia interaction. This parallel reduction in both plasma and urinary adenine suggests a broader systemic shift in adenine handling, possibly involving renal transport processes. Recent studies have postulated that SGLT2 inhibition may influence the activity of other renal transporters, including solute carriers involved in urate, amino acid, and vitamin transport, as well as organic anion transporters on the basolateral membrane (Billing et al., 2024; Gyimesi et al., 2020; Heerspink et al., 2021). SGLT2 inhibition may indirectly modulate the expression or function of these ancillary transporters that handle purine derivatives or related metabolites (Billing et al., 2024; Novikov et al., 2019; Suijk et al., 2022). Understanding the interplay between SGLT2 inhibition and purine metabolism at the level of renal transport may yield new insights into the pathogenesis and treatment of DKD. In our previous work, adenine was found to stimulate matrix production in tubular cells by activating the mTOR signaling pathway (Sharma et al., 2023). Kidney biopsies from participants with T2D and healthy controls showed that T2D is associated with upregulation of the mTORC1 pathway in each tubular segment of the nephron (Schaub et al., 2023; Sharma et al., 2023). SGLT2 inhibition is thought to attenuate aberrant mTOR signaling and restore proper mTOR cycle regulation through the reduction of blood glucose levels (Schaub et al., 2023; Sciarretta et al., 2018). As such, this upregulation of urine adenine was subsequently reduced with SGLT2 inhibition (Sharma et al., 2023), which we were able to replicate in plasma adenine within the current analysis. Although more work is required to delineate the potential interplay between adenine, methionine, and the mTOR pathway in diabetic kidney disease, our data lend support that hyperglycemia drives glucose metabolism to the mTOR cycle and SGLT2 inhibition mitigates the effects of the mTOR pathway by way of adenine, methionine, and cysteine.

Similar to our findings in plasma, in our prior work, we found that hyperglycemia augmented grouped urine amino acid pathways, including BCAAs, aromatic amino acids, and arginine biosynthesis (Kugathasan et al., 2024). Notably, we found that empagliflozin was associated with an increase in urine BCAAs under clamped hyperglycemia (Kugathasan et al., 2024). Although no changes to plasma BCAAs were demonstrated in response to empagliflozin, we observed an increase in plasma BCAAs in response to acute hyperglycemia. It appears that, while reduction–oxidation balance plays a role in modulating BCAA expression, the primary drivers are hyperglycemia and severe insulin resistance (Wang et al., 2011). These metabolic conditions may have a greater impact on BCAAs, suggesting that the altered glucose and insulin dynamics in such states override the effects of reduction–oxidation balance on BCAA metabolism. This provides insight into why an increase in BCAA was observed under ambient hyperglycemia with or without empagliflozin treatment, and not observed under the euglycemic clamp. Moreover, in our prior work, we investigated other plasma metabolites involved in fatty acid metabolism, and we observed similar findings, particularly the increase in alanine, aspartate, and glutamate metabolism during acute hyperglycemia, prior to treatment (Liu et al., 2021). While we evaluated a range of different metabolites, the results reinforced the same overall conclusion. Furthermore, we did not observe an increase in plasma kynurenine levels as seen in urine (Kugathasan et al., 2024); however, we observed an increase under clamped euglycemia and clamped hyperglycemia with treatment, inconsistent with the hypothesized potential protective effect of empagliflozin against oxidative stress (Marfella et al., 2001) and inflammation (Wang et al., 2015) in this patient population. Overall, the complex interplay between glucose metabolism, insulin resistance, and amino acid regulation should be further investigated, especially in patients with T1D.

This post hoc exploratory analysis has several limitations. Given that this analysis was exploratory and post hoc, and that the intervention trial lacked a control group, causality should not be directly inferred from these associations. Additionally, the small sample size and the relatively short 8‐week treatment period limit the ability to generalize the long‐term effects of SGLT2 inhibition to the broader population. Due to the limited duration of the glycemic clamp, the observed effects of hyperglycemia are confined to acute and ambient changes in metabolism. While the changes in plasma metabolite levels likely reflect metabolic alterations in the kidney, it remains possible that these findings may be attributed to broader systemic changes in energy metabolism, as we did not conduct kidney biopsies to confirm structural and functional alterations in renal mitochondria. Moreover, while our metabolomics data suggest altered amino acid metabolism, we did not perform metabolic flux analysis, which would provide direct evidence of amino acid utilization in energy metabolism. Therefore, future studies using stable isotope tracer‐based flux analysis are warranted to mechanistically validate the metabolic roles of amino acids suggested by our findings. A key limitation of this study is the restricted coverage of metabolites, which constrains the depth of mechanistic insight that can be drawn from pathway‐level analyses. The targeted metabolomics platform used in this work quantified a focused panel of amino acids and related metabolites but did not include key intermediates in many of the metabolic pathways identified by enrichment tools such as MetaboAnalyst. As a result, pathway assignments, including those related to folate metabolism, glyoxylate metabolism, BCAA metabolism, and biosynthetic pathways for essential amino acids, may not reflect actual biochemical activity in vivo and should be interpreted with caution. Notably, intermediates such as alpha‐ketoglutarate, which play a central role in transamination and TCA cycle integration, were not quantified. These computational pathway outputs are based on statistical overlap with canonical pathways rather than biological plausibility or direct evidence of pathway flux. Finally, it is possible that some of the changes observed in this study could be linked to variations in total daily insulin during the study period. However, despite a 16.4% reduction in total daily insulin following drug initiation to mitigate hypoglycemia risk (Cherney, Perkins, Soleymanlou, Maione, et al., 2014; Perkins et al., 2014), such adjustments are a standard part of care for individuals with T1D who are also receiving other antidiabetic treatments.

## 5 PERSPECTIVES AND SIGNIFICANCE

Our findings highlight the metabolic changes induced by SGLT2 inhibition and acute hyperglycemia, particularly in the context of plasma amino acid metabolism. The increase in amino acid metabolism was mitigated by empagliflozin treatment and likely served as protection against dysfunctional systemic and renal metabolic alterations involving energy utilization and regulation. This provides further evidence for the concept of a “pseudo‐fasting state” induced by SGLT2 inhibition (Ferrannini et al., 2014; Ferrannini, Baldi, et al., 2016; Hesp et al., 2020), to optimize energy substrate efficiency, particularly in energy‐demanding organs like the heart and kidneys. Given the known cardiovascular and kidney risks in individuals with T1D, this adaptive metabolic response could have potential therapeutic implications. By improving energy utilization and supporting mitochondrial function, SGLT2 inhibition may offer protective benefits against diabetic complications, including cardiovascular disease and kidney failure. These findings emphasize the importance of further investigating the long‐term impact of SGLT2 inhibitors on metabolic processes and organ function in T1D.

## AUTHOR CONTRIBUTIONS

All authors contributed to the collection of the data and data interpretation. L.K. performed the statistical analysis and wrote the first draft of the manuscript. D.Z.I.C was the principal investigator involved in the ATIRMA study design. All authors provided critical revision for important intellectual content and approved the final version of the manuscript for submission. The corresponding author K.S. takes full responsibility for the work and/or conduct of the study, had access to the data, and controlled the decision to publish.

## FUNDING INFORMATION

The current work was supported by an investigator‐initiated grant from Boehringer Ingelheim and Eli Lilly and Company. KS is supported by the NIDDK 2U01DK114920‐06, a Veterans Affairs Merit Award 2I01BX001340‐09A1, and an NHLBI subaward via New York University and NIH SBIR Subaward to SygnaMap (1R43DK130732‐01A1). DZIC is supported by a Department of Medicine, University of Toronto Merit Award and receives support from CIHR, Diabetes Canada, and the Heart and Stroke Richard Lewar Centre of Excellence. DZIC is also the recipient of a CIHR‐KFOC Teams Grant Award.

## CONFLICT OF INTEREST STATEMENT

KS has served as a consultant and received honoraria from Boehringer Ingelheim, Janssen, and Sanofi. KS has received research support from Boehringer Ingelheim. DZIC has received honoraria from Boehringer Ingelheim‐Lilly, Merck, AstraZeneca, Sanofi, Mitsubishi‐Tanabe, Abbvie, Janssen, Bayer, Prometic, BMS, Maze, Gilead, CSL‐Behring, Otsuka, Novartis, Youngene, Lexicon, and Novo‐Nordisk and has received operational funding for clinical trials from Boehringer Ingelheim‐Lilly, Merck, Janssen, Sanofi, AstraZeneca, CSL‐Behring, and Novo‐Nordisk. VSS has received conference and travel support from Merck Canada. BAP has received speaker honoraria from Abbott, Bayer, Insulet, Dexcom, Medtronic, Novo Nordisk, and Sanofi; has received research grant support from Novo Nordisk and BMO Bank of Montreal; and serves as a consultant for Abbott, Insulet, Dexcom, Sanofi, Vertex, and Nephris. LK, NR, SD, TF, and ET do not have any disclosures relevant to this paper to report.

## ETHICS STATEMENT

The local Research Ethics Board at the University Health Network (Toronto, Canada) approved the protocol, and all subjects gave informed consent prior to the start of procedures. The study was conducted according to the International Conference on Harmonization on Good Clinical Practice.

## Supporting information


Tables S1–S4.

## Data Availability

Source data for this study (https://doi.org/10.1161/CIRCULATIONAHA.113.005081) are not publicly available due to privacy or ethical restrictions. The source data are available to verified researchers upon request by contacting the corresponding author. The authors confirm that the data supporting the findings of this study are openly available in figshare at https://figshare.com/s/d3cedb654cf80a2d8766.
