# Supplementary material for: Metabolic shifts in plasma amino acids and related metabolites in response to SGLT2 inhibition and hyperglycemia in type 1 diabetes
Source: Physiol Rep. 2025 Aug 22;13(16):e70465. doi: 10.14814/phy2.70465 (PMC12371268; doi:10.14814/phy2.70465)
Supplement: Supplementary file 1 — Tables S1–S4. [file PHY2-13-e70465-s001.docx]

**SUPPLEMENTAL MATERIAL**

**Supplemental Table S1.** Least squares mean values (and standard error) of log transformed individual metabolites in plasma at each timepoint.

| **Metabolite** | **Visit 3** | **Visit 4** | **Visit 12** | **Visit 13** |
| --- | --- | --- | --- | --- |
| 5-Methylthioadenosine | -3.6314 (0.189) | -3.5088 (0.189) | -3.5542 (0.1917) | -3.7735 (0.1917) |
| Acetyl-Lysine | -0.6314 (0.07326) | -0.6834 (0.07326) | -0.5101 (0.07353) | -0.4900 (0.07353) |
| Adenine | -5.6346 (0.158) | -5.3695 (0.158) | -5.2082 (0.1594) | -5.5007 (0.1594) |
| Alanine | 5.7418 (0.04563) | 5.9271 (0.04563) | 5.6039 (0.04606) | 5.7665 (0.04606) |
| Arginine | 4.0847 (0.04562) | 4.1998 (0.04562) | 4.0586 (0.04611) | 4.1215 (0.04611) |
| Asparagine | 3.6567 (0.03605) | 3.7485 (0.03605) | 3.7036 (0.03637) | 3.7484 (0.03637) |
| Carnitine | 7.6684 (0.05129) | 7.6392 (0.05129) | 7.6427  (0.05147) | 7.6859 (0.05147) |
| Citrulline | 2.7420 (0.05875) | 2.7544 (0.05875) | 2.8902 (0.05913) | 2.9041 (0.05913) |
| Cystathionine | 3.2301  (0.1646) | 3.1360  (0.1646) | 3.3654  (0.1654) | 3.4204  (0.1654) |
| Cysteine | 3.6074 (0.08571) | 3.5365 (0.08571) | 3.7586 (0.08642) | 3.8217 (0.08642) |
| Glutamic Acid | 3.8781 (0.07211) | 3.8062 (0.07211) | 3.8545 (0.0727) | 3.8647 (0.0727) |
| Glutamine | 6.1869 (0.02661) | 6.2605 (0.02661) | 6.1737 (0.02682) | 6.2138 (0.02682) |
| Glycine | 5.6476 (0.05502) | 5.6943 (0.05502) | 5.6928 (0.05535) | 5.7413 (0.05535) |
| Glycyl-histidine | -4.0426 (0.1528) | -4.1941 (0.1528) | -4.2199 (0.1539) | -4.2167 (0.1539) |
| Histidine | 4.2033 (0.02738) | 4.2197 (0.02738) | 4.1818 (0.02754) | 4.1901 (0.02754) |
| Isoleucine | 3.8724 (0.143) | 4.1196 (0.143) | 3.8396 (0.1451) | 4.1073 (0.1451) |
| Kynurenine | 4.0407 (0.04724) | 4.1455 (0.0476) | 4.1809 (0.04763) | 4.2561 (0.04763) |
| L-alpha-Aminobutyric acid | 1.4099 (0.05628) | 1.4952 (0.05628) | 1.6665 (0.05674) | 1.6772 (0.05674) |
| Lysine | 5.0132 (0.03474) | 5.0661 (0.03474) | 5.0573 (0.03502) | 5.0905 (0.03502) |
| Methionine | 2.8052 (0.03502) | 3.0048 (0.03502) | 2.8215 (0.03545) | 2.9798 (0.03545) |
| Mono-Methyl Arginine | 0.2781 (0.1010) | 0.3029 (0.1010) | -0.1250  (0.1016) | -0.02125 (0.1016) |
| Nicotinic acid | -3.6215 (0.1883) | -3.6914 (0.1883) | -3.5004 (0.189) | -3.6937 (0.189) |
| Ornithine | 3.7158 (0.06559) | 3.7784 (0.06559) | 3.6721 (0.06612) | 3.789 (0.06612) |
| Phenylalanine | 0.2055 (0.05483) | 0.2306 (0.05483) | 0.134 (0.05524) | 0.07573 (0.05524) |
| Pipecolate | 1.338 (0.09589) | 1.3527 (0.09589) | 1.5008 (0.09633) | 1.458 (0.09633) |
| Proline | 5.4366 (0.06464) | 5.5839 (0.06464) | 5.4391 (0.06502) | 5.6227 (0.06502) |
| S-adenosylhomocysteine | -3.7912 (0.2335) | -3.9562 (0.2335) | -3.6109 (0.2369) | -3.9829 (0.2369) |
| S-adenosylmethionine | -0.1988 (0.03298) | -0.2111 (0.03298) | -0.211 (0.03303) | -0.2297 (0.03303) |
| SDMA+ADMA | 0.2280 (0.1333) | 0.1320  (0.1333) | 0.3819 (0.1342) | 0.4267 (0.1342) |
| Serine | 4.7275 (0.03462) | 4.8677 (0.03462) | 4.7658 (0.03497) | 4.8657 (0.03497) |
| Taurine | 5.4799  (0.04842) | 5.3647 (0.04842) | 5.5691 (0.04886) | 5.4941 (0.04886) |
| Threonine | 4.4773 (0.04955) | 4.6268 (0.04955) | 4.3722 (0.04992) | 4.5289 (0.04992) |
| Tryptophan | 3.3995 (0.04199) | 3.4382 (0.04199) | 3.351 (0.04216) | 3.365 (0.04216) |
| Tyrosine | 3.6754 (0.03409) | 3.7161 (0.03409) | 3.7345 (0.03434) | 3.7099 (0.03434) |
| Valine | 4.6084 (0.07212) | 4.616 (0.07212) | 4.6008 (0.07237) | 4.6506 (0.07237) |

*Visit 3* – Clamped euglycemia, baseline.

*Visit 4* – Clamped hyperglycemia, baseline.

*Visit 12* – Clamped euglycemia, post-treatment.

*Visit 13* – Clamped hyperglycemia, post-treatment.

**Supplemental Table S2.** Retention times of selected metabolites analyzed by LC-MS.

| **Metabolite name** | **Retention Time** |
| --- | --- |
| Nicotinic acid | 1.32 |
| 5-Methylthioadenosine | 2.12 |
| Adenine | 2.86 |
| Tryptophan | 3.21 |
| Creatinine | 3.26 |
| Kynurenine | 3.35 |
| Phenylalanine | 3.29 |
| Tyrosine | 3.35 |
| Isoleucine | 3.42 |
| Methionine | 3.48 |
| L-alpha + gamma Aminobutyric acid | 3.63 |
| Glutamic Acid | 3.59 |
| Serine | 3.66 |
| Alanine | 3.71 |
| Pipecolate | 3.75 |
| Threonine | 3.63 |
| Glycine | 3.71 |
| Asparagine | 3.75 |
| Proline | 3.82 |
| Glutamine | 3.85 |
| S-Adenosyl homocysteine | 3.84 |
| Valine | 4.16 |
| Arginine | 4.2 |
| Cystine | 4.13 |
| Ornithine | 4.21 |
| Lysine | 4.23 |
| Histidine | 4.26 |
| Glycyl-histidine | 4.44 |
| S-Adenosyl Methionine | 4.64 |
| Cystathionine | 0.6 |
| Taurine | 2.95 |
| Acetyl-Lysine | 3.89 |
| Citrulline | 3.92 |
| Mono-Methyl Arginine | 4.17 |
| SDMA+ADMA | 4.37 |
| Carnitine | 4.13 |

**Supplemental Table S3. Specifics of the commercial standards.**

| **Metabolite Name** | **CAS Number** | **Vendor** | **Purity** |
| --- | --- | --- | --- |
| Nicotinic acid | 59‑67‑6 | Sigma‑Aldrich | ≥99% |
| 5‑Methylthioadenosine (MTA) | 2457‑80‑9 | Sigma‑Aldrich | ≥98% |
| Adenine | 73‑24‑5 | Sigma‑Aldrich | ≥99% |
| Creatinine | 60‑27‑5 | Sigma‑Aldrich | ≥98% |
| Kynurenine | 2922‑83‑0 | Sigma‑Aldrich | ≥98% |
| γ‑Aminobutyric acid (GABA) | 56‑12‑2 | Sigma‑Aldrich | ≥99% |
| α‑Aminobutyric acid | [1492-24-6](https://www.sigmaaldrich.com/US/en/product/sigma/a1879?srsltid=AfmBOoqrP854QWQDuhgtsMF11Fer4O5AQuNBu7FOM4N5UR6TVgH5Q8gh) | Sigma‑Aldrich | ≥99% |
| Pipecolate | 273‑67‑6 | Sigma‑Aldrich | ≥99% |
| Glutamine | 56‑85‑9 | Sigma‑Aldrich | ≥99% |
| S‑Adenosyl homocysteine (SAH) | 979‑92‑0 | Sigma‑Aldrich | ≥98% |
| Ornithine | 70‑26‑8 | Sigma‑Aldrich | ≥99% |
| Glycyl-histidine | 3486-76-8 | Sigma‑Aldrich | ≥99% |
| S‑Adenosyl methionine (SAM) | 52248-03-0 | MedChemExpress | ≥99% |
| Cystathionine | 3035‑10‑9 | Sigma‑Aldrich | ≥90% |
| Taurine | 107‑35‑7 | Sigma‑Aldrich | ≥98% |
| Acetyl‑Lysine | [1946-82-3](https://www.sigmaaldrich.com/US/en/search/1946-82-3?focus=products&page=1&perpage=30&sort=relevance&term=1946-82-3&type=cas_number) | Sigma‑Aldrich | ≥98% |
| Citrulline | 372‑75‑8 | Sigma‑Aldrich | ≥98% |
| Mono‑Methyl Arginine (MMA) | 53308-83-1 | Sigma‑Aldrich | ≥99% |
| Symmetric Dimethyl-L-arginine (SDMA) | 1266235-58-8 | Sigma‑Aldrich | ≥99% |
| Asymmetric Dimethyl-L-arginine (ADMA) | [220805-22-1](https://www.sigmaaldrich.com/US/en/search/220805-22-1?focus=products&page=1&perpage=30&sort=relevance&term=220805-22-1&type=cas_number) | Sigma‑Aldrich | ≥99% |
| Carnitine | 541‑15‑1 | Sigma‑Aldrich | ≥99% |
| Amino acids | MSK-A2-US-1.2 | CIL, Inc. | ≥98% |

**Supplemental Table S4.** Plasma metabolite pathway compositions determined by MetaboAnalyst 6.0.

| **Group** | **Metabolite** | **Hits** | **Total** | **FDR** |
| --- | --- | --- | --- | --- |
| Cysteine and methionine metabolism | 5-Methylthioadenosine  S-adenosylmethionine  Serine  Methionine  S-adenosylhomocysteine  Cysteine  L-alpha-Aminobutyric acid  Cystathionine | 8 | 33 | <0.0001 |
| One carbon pool by folate | Glycine  Methionine  S-adenosylmethionine  S-adenosylhomocysteine  Serine  Cystathionine  Cysteine | 7 | 26 | <0.0001 |
| Arginine biosynthesis | Glutamic acid  Arginine  Ornithine  Glutamine  Citrulline | 5 | 14 | <0.0001 |
| Glycine, serine and threonine metabolism | Serine  Glycine  Threonine  Cysteine  Cystathionine | 5 | 33 | 0.0056 |
| Valine, leucine and isoleucine biosynthesis | Threonine  Isoleucine  Valine | 3 | 8 | 0.0056 |
| Arginine and proline metabolism | Arginine  Proline  Glutamic acid  Ornithine  S-adenosylmethionine | 5 | 36 | 0.0065 |
| Glutathione metabolism | Glycine  Cysteine  Glutamic acid  Ornithine | 4 | 28 | 0.0174 |
| Alanine, aspartate and glutamate metabolism | Asparagine  Alanine  Glutamic acid  Glutamine | 4 | 28 | 0.0174 |
| Phenylalanine, tyrosine and tryptophan biosynthesis | Tyrosine  Phenylalanine | 2 | 4 | 0.0191 |
| Glyoxylate and dicarboxylate metabolism | Glutamic acid  Glutamine  Serine  Glycine | 4 | 32 | 0.0231 |
| Nitrogen metabolism | Glutamic acid  Glutamine | 2 | 6 | 0.0381 |
| Phenylalanine metabolism | Tyrosine  Phenylalanine | 2 | 8 | 0.0588 |
| Taurine and hypotaurine metabolism | Cysteine  Taurine | 2 | 8 | 0.0588 |

MetaboAnalyst 6.0 software was used to identify the compositions of metabolic pathways that sufficiently represented (FDR ≤ 0.1) the 35 plasma metabolites.

Principal component analysis was conducted to reduce dimensionality. Available metabolites within each pathway were projected onto the first principal component to represent the pathway.

*Hits* – the number of metabolites included in the pathway that were measured in this analysis.

*Total* – total number of metabolites in the pathway generated by the KEGG (Kyoto encyclopedia of genes and genomes) database.

*FDR* – false discovery rate *p* value
